# Audiovisual Learning in Dyslexic and Typical Adults: Modulating Influences of Location and Context Consistency

**DOI:** 10.3389/fpsyg.2021.754610

**Published:** 2021-10-28

**Authors:** Simone L. Calabrich, Gary M. Oppenheim, Manon W. Jones

**Affiliations:** School of Psychology, Bangor University, Bangor, United Kingdom

**Keywords:** cross-modal binding, looking-at-nothing, paired associate learning, visual-phonological associations, webcam-based eye-tracking, developmental dyslexia (DD), episodic memory, reading

## Abstract

Learning to read involves efficient binding of visual to auditory information. Aberrant cross-modal binding skill has been observed in both children and adults with developmental dyslexia. Here, we examine the contribution of episodic memory to acquisition of novel cross-modal bindings in typical and dyslexic adult readers. Participants gradually learned arbitrary associations between unfamiliar Mandarin Chinese characters and English-like pseudowords over multiple exposures, simulating the early stages of letter-to-letter sound mapping. The novel cross-modal bindings were presented in consistent or varied locations (i.e., screen positions), and within consistent or varied contexts (i.e., co-occurring distractor items). Our goal was to examine the contribution, if any, of these episodic memory cues (i.e., the contextual and spatial properties of the stimuli) to binding acquisition, and investigate the extent to which readers with and without dyslexia would differ in their reliance on episodic memory during the learning process. Participants were tested on their ability to recognize and recall the bindings both during training and then in post-training tasks. We tracked participants’ eye movements remotely with their personal webcams to assess whether they would re-fixate relevant empty screen locations upon hearing an auditory cue—indicative of episodic memory retrieval—and the extent to which the so-called “looking-at-nothing behavior” would modulate recognition of the novel bindings. Readers with dyslexia both recognized and recalled significantly fewer bindings than typical readers, providing further evidence of their persistent difficulties with cross-modal binding. Looking-at-nothing behavior was generally associated with higher recognition error rates for both groups, a pattern that was particularly more evident in later blocks for bindings encoded in the inconsistent location condition. Our findings also show that whilst readers with and without dyslexia are capable of using stimulus consistencies in the input—both location and context—to assist in audiovisual learning, readers with dyslexia appear particularly reliant on consistent contextual information. Taken together, our results suggest that whilst readers with dyslexia fail to efficiently learn audiovisual binding as a function of stimulus frequency, they are able to use stimulus consistency—aided by episodic recall—to assist in the learning process.

## Introduction

Quickly binding visual forms to phonological forms is a fundamental skill in the initial stages of grapheme-phoneme learning, providing a foundation for the later development of integrated visual-phonological representations that are crucial for skilled reading. Most children are able to convert written letters and words into sounds effortlessly, and later retrieve them as a single audiovisual unit, eventually becoming proficient readers. However, some struggle to form novel audiovisual mappings, a difficulty that can persist well into adulthood (Blau et al., 2009; Jones et al., 2013b, 2018). Readers with developmental dyslexia exhibit indications of less-integrated grapheme-phoneme representations (Blau et al., 2009, 2010; Blomert, 2011; Warmington and Hulme, 2012; Aravena et al., 2013, 2018; Žarić et al., 2015), a deficit owing in part to their comparatively poorer cross-modal binding skills (Aravena et al., 2013; Jones et al., 2013b, 2018; Žarić et al., 2015; Albano et al., 2016; Toffalini et al., 2018, 2019; Garcia et al., 2019). Despite the well-known link between audiovisual integration and ultimate reading attainment, the cognitive mechanisms underlying typical and atypical cross-modal binding ability are not yet fully understood. Here, we examine how adults with dyslexia and typical readers may differ in their reliance on episodic memory cues as they acquire novel cross-modal bindings that vary in location-related and contextual consistency over the course of the learning process.

Learning to read requires establishing new representations in memory: not only separate representations for novel visual/orthographic and phonological forms, but also correspondences between them. A commonly used task to tap the acquisition of novel visual-phonological mappings is cross-modal *paired associate learning* (PAL; e.g., Warmington and Hulme, 2012; Wang et al., 2017; Jones et al., 2018; Calabrich et al., 2021), in which participants must learn that a given visual symbol is associated with a particular phonological sequence (typically a pseudoword). This learning process is thought to emulate the associative mechanisms underpinning grapho-phonological mappings in the early stages of literacy development (Hulme et al., 2007; Warmington and Hulme, 2012). An extensive body of research demonstrates that readers with dyslexia are generally more error prone on such cross-modal PAL tasks, relative to typical readers (Messbauer and de Jong, 2003; Warmington and Hulme, 2012; Litt and Nation, 2014; Wang et al., 2017; Jones et al., 2018; Toffalini et al., 2018), and, crucially, performance on PAL tasks correlates with individual differences in reading skill (Hulme et al., 2007; Warmington and Hulme, 2012). In particular, visual-verbal PAL ability is a unique predictor of both word recognition and non-word reading (Warmington and Hulme, 2012).

Whilst PAL tasks are useful in showing the relationship between visual-verbal learning and reading ability, such paradigms do not typically elucidate the learning mechanisms that distinguish good and poorer performance in PAL and reading. However, in other learning contexts, the ability to track simple statistics, such as stimulus repetition and sequences is a strong predictor of reading ability (Ahissar, 2007), and poorer readers are liable to forget previous exposures to perceptual stimuli (Jaffe-Dax et al., 2015, 2016, 2017), potentially leading to “noisier” processing of a current stimulus. We can therefore reasonably extrapolate that statistical tracking, implicating episodic memory and associated decay, may play an important role in determining the effectiveness with which audiovisual associations can be created and established over repeated exposures. Indeed, learning audiovisual stimuli requires accurate encoding of temporal and spatial characteristics in order to appropriately bind visual and phonological features and to create a composite representation. Temporal and spatial properties, commonly encoded in episodic memory, share patterns of neural activity, and can be used as cues to aid memory retrieval when required (Tulving, 1972; El-Kalliny et al., 2019). In the context of language, episodic memory of the context in which a word is encountered plays an important role in acquisition (Stark and Stark, 2016). Through repetition and rehearsal, representations become gradually less episodic and more abstract, representative of an amalgam of consistent stimulus properties, with the result that specific episodic details, such as spatial and temporal properties, become less and less relevant (Squire and Zola, 1998; Stark and Stark, 2016). In literacy acquisition, this process also entails a gradual increase in automatization of print reading, such that phonology is eventually accessed automatically and without recourse to an effortful retrieval process, implicating episodic memory resources (LaBerge and Samuels, 1974; Ehri and Saltmarsh, 1995; Ehri, 2005; Jones et al., 2013a,b, 2018).

Recently, Jones et al. (2018) examined the role of statistical learning mechanisms and episodic memory in the context of a PAL task, in which groups of readers with dyslexia and typically reading adults learned to associate a sequence of unfamiliar characters (i.e., Mandarin Chinese characters) with consistently paired pseudowords. As participants attempted to retrieve each target’s corresponding pseudoword, their eye movements were tracked on the now-blank screen to examine whether they consulted the spatial location of the target item in order to support retrieval. Such “looking-at-nothing” behavior would imply re-activation of integrated memory representations: re-activating one of the target features, such as its phonological representation, may automatically drive the memory system to re-activate other features as well, including its visuospatial location, mechanistically or epiphenomenally producing eye movements toward that location (Altmann, 2004; Ferreira et al., 2008; Scholz et al., 2011; Johansson and Johansson, 2020; Kumcu and Thompson, 2020) when rebinding the multiple features again. Although such looking-at-nothing movements can suggest both successful memory encoding and reactivation in the earliest stages of learning, they also become less frequent as learners consolidate a memory representation, presumably abstracting away such details (Spivey, 2007; Scholz et al., 2011). For readers with dyslexia in Jones et al. (2018) study, fixating a target’s former location within the current trial was associated with greater recall accuracy (which nevertheless stayed well below par, compared with the typical reader group), and fixating a distractor’s former location was associated with lesser recall accuracy, both compared to a no-fixation baseline. For typical readers, in contrast, fixating a target’s former location within the current trial was only associated with greater recall accuracy when it had also appeared in the same location in a previous trial. Thus, whilst readers with dyslexia showed sensitivity to location information for only the current trial, typical readers showed a sensitivity to longer-range statistical regularities over multiple exposures. This pattern suggests that typical readers use spatial location as a cue to retrieve such bindings, even when location information is task irrelevant, and this ability may specifically be impaired in readers with dyslexia (Jones et al., 2013b, 2018; Albano et al., 2016; Toffalini et al., 2018).

Audiovisual learning is therefore modulated by the statistical sensitivity and associated episodic memory usage that individuals of different reading abilities bring to bear on the task. However, we are yet to discover how specific stimulus configurations during learning affect learning efficiency in dyslexic and typical readers. In general, presenting multiple items in a temporally adjacent format increases the association between these items (El-Kalliny et al., 2019). However, isolating and retrieving *individual* memories encoded in temporal proximity can only succeed if the distinct memories were separated in neural space during encoding (Sheehan et al., 2018; El-Kalliny et al., 2019). In other words, our ability to discriminate between different past experiences that share similar features largely depends on the brain’s capacity to store *distinct* activity patterns to represent *each* of these experiences (Madar et al., 2019). Readers with dyslexia have been shown to benefit from having novel cross-modal bindings presented in a fixed temporal order (Toffalini et al., 2018), but, to the best of our knowledge, there is no comprehensive study of how stimulus configurations during learning affect typical and dyslexic readers’ capacity to learn reading-related items. This is an important next step, since dyslexic readers’ reduced ability to create stable representations over multiple exposures is plausibly related to their inability to identify an item as distinct from other items presented in temporal and spatial proximity.

In the present study, we examine whether specific statistical properties of stimulus exposures differentially affect learning in adults with and without developmental dyslexia. To this end, we designed a PAL task (adapted from Jones et al., 2018), in which we manipulated the consistency of the spatial and contextual stimulus properties during encoding. We created arbitrary associations between monosyllabic pseudowords—following English phonotactics (e.g., /gᴐp/)—and Mandarin Chinese characters (e.g., **⽇**). Our participants were unfamiliar with both the visual and auditory stimuli, thus ensuring an arbitrary relationship between these visual-verbal bindings, and simulating the early stages of orthography-to-phonology mapping.

In terms of accuracy, we predicted that, compared with typical readers, readers with dyslexia would show generally higher error rates, and a shallower function of learning (Messbauer and de Jong, 2003; Aravena et al., 2013; Jones et al., 2013b, 2018; Albano et al., 2016; Toffalini et al., 2018, 2019; Garcia et al., 2019). Further, whilst we predicted that consistently presenting targets in the same spatial location and/or in the context of the same alternatives would generally decrease error rates, we suspected that these consistency effects would disproportionately benefit readers with dyslexia: though previous work indicates that readers with dyslexia are less likely to track single-feature statistics (e.g., location) over multiple exposures (Jones et al., 2013b, 2018; Toffalini et al., 2018), providing *both* spatial (i.e., item screen location) *and* contextual consistencies (i.e., item co-occurrences) might prove particularly advantageous to help impaired readers bootstrap degraded representations/poorer retrieval of individual items. Indeed, readers with dyslexia are known to engage in chunking strategies such as whole word memorization in order to avoid phonological sequencing, which is problematic in dyslexia (Ullman and Pullman, 2015).

To consider the possible role of implicit memory retrieval, we estimated participants’ reference to episodic detail *via* a looking-at-nothing paradigm. During the main training and recognition task, we made novel use of webcam-based technology (WebGazer.js: Papoutsaki et al., 2016) to remotely track participants’ eye movements as they viewed a blank screen immediately after hearing an auditory cue. Even though the use of webcam-based eye tracking in behavioral science is still in its infancy, previous investigations have demonstrated the method’s suitability to detect fixations reliably and to replicate in-lab findings with minimal reduction in data quality (Bott et al., 2017; Semmelmann and Weigelt, 2018). With this approach, we sought to ascertain whether looks to relevant blank screen locations would modulate recognition accuracy. Following previous work (Jones et al., 2018), we predicted that readers with dyslexia would have a stronger tendency to make errors following fixations to blank screen locations previously occupied by distractor items. We also expected repetition to diminish the link between accuracy and looking-at-nothing behaviors for all participants, reflecting direct access to increasingly abstracted memory representations (Richardson and Spivey, 2000; Ferreira et al., 2008; Scholz et al., 2011; Wantz et al., 2016). Finally, our factorial manipulation allows us to consider higher-order interactions, but it is challenging to derive and evaluate specific predictions for such interactions, and robustly assessing such interactions would require more power than our study provides (Button et al., 2013); as a compromise, we note such interactions but consider them primarily as invitations for future research.

In addition to the main training and recognition task, we collected three additional measures of item learning. We added (1) cued-recall trials at regular intervals in the main training task to test participants’ ability to recall and verbalize the specific pseudoword associated with a given character. Moreover, to probe participants’ longer-term memory, we tested participants’ ability to (2) recall, and (3) recognize the bindings in two separate tasks administered approximately 10 min after the main task. This approach allowed us to assess whether the episodic memory effects of spatial and contextual cues carried over and differentially modulated longer term retention of the bindings for the two reading groups. Due to the gradual consolidation process engendered by repeated exposures, we predict that performance in the subsequent post-training cued-recall and recognition tests would be less strongly modulated by episodic memory cues. We also predicted overall higher error rates in recall than in recognition, given that recognition is wont to succeed even when recall fails (Tulving, 1982).

## Materials and Methods

### Participants

Thirty-five readers with dyslexia (age: *M* = 28.17, *SD* = 7; 23 females) and thirty-five typical readers (age: *M* = 23.55, *SD* = 6.14; 19 females) were tested remotely. All participants were native speakers of British English, recruited through Bangor University and Prolific.^1^ A similar level of education was reported in both groups (dyslexia: *M* = 15.8 years, *SD* = 2.37; typical: *M* = 14.8 years, *SD* = 2.11; *p* = 0.09), and none of the participants reported any history of psychiatric and/or neurological diseases, visual and/or hearing impairments, or any other risk factors. Group membership (i.e., typical reader or individual with dyslexia) was confirmed *via* a battery of literacy tests. All participants provided informed consent, were naïve to the purpose of the experiment, and were unfamiliar with the experimental stimuli. Participants received course credits or payment for their time. The experiment was approved by Bangor University’s Ethics Committee.

### Literacy and General Cognitive Ability Measures

Participants’ group membership was validated *via* a battery of eight short tests: (1) *Adult Reading Questionnaire* (ARQ, Snowling et al., 2012); (2) word reading efficiency and (3) phonemic decoding efficiency subscales of the *Test of Word Reading Efficiency* (TOWRE, Torgesen et al., 1999); (4) letter and (5) digit versions of the *Rapid Automatized Naming* (RAN) subtest from the *Comprehensive test of Phonological Processing* (CTOPP, Wagner et al, 1999); (6) *Similarities* subtest from the *Wechsler Adult Intelligence Scale* (WAIS, Wechsler, 1981) as an index of verbal intelligence quotient (IQ); (7) *Matrix Reasoning* from the *Wechsler Abbreviated Scale of Intelligence* (WASI, Wechsler, 1999) as an index of non-verbal IQ; and (8) computerized forward and backward digit span tests in which participants first saw sequences of digits and were then prompted to type the digits in the same or reverse order. Tests 1–5 were administered shortly before the main training and recognition task, whereas the remaining were administered immediately after the main task.

### Stimuli

Thirty-six consonant-vowel-consonant (CVC) pseudowords (e.g., /gᴐp/) were arbitrarily matched to thirty-six Mandarin Chinese characters (e.g., **⽇**), as in Jones et al. (2018). The pseudowords followed English phonotactic rules and were generated with Wuggy (Keuleers and Brysbaert, 2010), a multilingual pseudoword generator. The auditory stimuli were recorded by a female native speaker of British English and digitized at 44.1 kHz on Praat (Boersma and Weenink, 2021). Each Mandarin Chinese character was consistently presented with the same CVC pseudoword over the course of the experiment.

#### Procedure

The experiment was programmed and deployed online on Gorilla Experiment Builder (Anwyl-Irvine et al., 2020). It included three tasks, presented in the same order to all participants: (1) training, *via* a six-block recognition task with interspersed cued-recall trials; (2) a single-block cued-recall test; and (3) a single-block recognition test.

Access to the experiment was restricted to desktop and laptop users only; mobile phones and tablets were disallowed. Participants were instructed to wear earphones or headphones, to place their computers on a desk, and to do the tasks individually in a quiet and well-lit room. To minimize distraction and correct for varying screen sizes and resolutions, participants were prompted to activate the full screen mode on their computers before proceeding to the experimental tasks. On average, participants sat 546.03 mm (*SD* = 101.02) from their computer screens as estimated by the Virtual Chinrest task (Li et al., 2020). The entire testing session lasted approximately 130 min, including background tests, experimental tasks, and calibrations. A time limit of 180 min automatically rejected any participants exceeding this threshold.

Eye-tracking measures were assessed *via* WebGazer.js (Papoutsaki et al., 2016) with an ideal sampling rate of approximately 60 Hz, dependent on each participant’s monitor’s refresh rate (Anwyl-Irvine et al., 2020). Before each task, participants completed a 5-point calibration procedure. A series of pictorial instructions demonstrated appropriate head position during calibration and experimental tasks. Failure to calibrate at least one of the points (i.e., if the estimate for a point was too close to another) resulted in an automatic repetition of the calibration procedure. To account for participants’ potential head drift and body repositioning, re-calibration was performed in the middle of each experimental block (i.e., after 18 trials), and before the onset of each new block in training. Eye-tracking estimates with face confidence values (i.e., a score ranging from 0 to 1 estimating the webcam-based eye-tracking machine learning model’s confidence level in detecting a human face) lower than 0.5 were excluded from the analyses. In the two post-training tests, eye-tracking measures were recorded for exploratory purposes only and are not reported here.

##### Training: recognition (with interspersed cued-recall trials)

**FIGURE 1:**
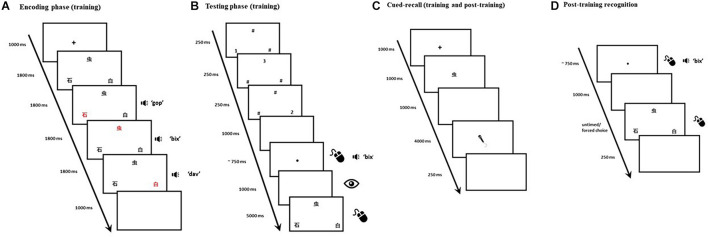
Panels **(A,B)** depict the timeline of a single trial in the main training and recognition task. The encoding phase **(A)** was immediately followed by backward masking and then by the testing phase **(B)**. Panel **(C)** depicts the timeline of a single cued-recall trial (both training and post-training). Panel **(D)** depicts the timeline of a single trial in the post-training recognition test. The eye depicts recording of onscreen fixations, the microphone depicts when a verbal response was expected, and the mouse illustrates when a click was expected.

Training emulates Calabrich et al. (2021) main paradigm, originally based on Jones et al. (2018) cued-recall paradigm. Each training trial consisted of an encoding phase and a testing phase. Each trial began with a 1,000-ms fixation cross, followed by three Mandarin Chinese characters presented in black on a white background. The three characters were displayed in triangle formation (see Figure 1A), each occupying 20 × 20 units of Gorilla Experiment Builder’s (Anwyl-Irvine et al., 2020) screen space. Each character’s color changed from black to red synchronously with auditory presentation of its corresponding pseudoword. The order in which character/pseudowords were highlighted/presented was fully counterbalanced across trials. At the end of this encoding phase, a 1,000-ms blank screen was followed by a visual backward masking phase: hash symbols and numbers, presented in pseudorandomized order, momentarily replaced the characters to minimize visible persistence (see Figure 1B). The onset of the testing phase was signaled by the appearance of a small black dot presented in the center of the screen. A click on the black dot would play the auditory cue that corresponded to the target (i.e., one of the three pseudowords from the encoding phase). If no clicks were detected within 10 s, the trial would terminate. The requirement to click the black dot had the secondary purpose of introducing an inconspicuous attention check: if, in three consecutive trials, no clicks had been detected, the participant would be automatically excluded from the experiment as this would constitute a strong indication that their computer had been left unattended mid-task. A 1,000-ms blank screen followed the black dot, during which participants’ eye movements were recorded. The three Mandarin Chinese characters then reappeared, and a mouse-click was expected: participants were instructed to select the character that corresponded to the auditory cue. In order to minimize auditory localization bias and encourage our participants to attend to both visual *and* auditory features of the stimuli, the characters’ screen position changed in two thirds of the trials once they reappeared in the testing phase. The characters remained on the screen for 5,000 ms, or until a mouse-click was detected, whichever occurred first. A 250-ms blank screen was presented, at which point the trial ended. A total of 216 trials were evenly distributed over 6 blocks. Block and trial presentation were randomized across participants to avoid order effects.

As in Calabrich et al. (2021), we orthogonally manipulated two binomial factors in the encoding phase: (1) *Location consistency*: whether a visual-phonological association was consistently presented in the same spatial location throughout the experiment, and (2) *Context consistency*: whether a visual-phonological association consistently co-occurred with the same items throughout the experiment. As a result, half of the 36 Mandarin Chinese characters were always presented in the same screen position across different trials (i.e., six items would only appear in the top middle, six in the bottom left, and six in the bottom right), whilst the other half could appear in any of three possible screen locations with equal probability. Similarly, half of the stimuli would always appear within a specific triplet (i.e., a target item along with the same two distractors, e.g., items A, B, and C would always be presented together in each occurrence—taking turns as targets and distractors across different trials—and would never co-appear with any other items over the course of the experiment), whilst the remaining would not have any fixed co-occurrences. For each participant, each binding was therefore assigned to one of four trial types: (1) *Inconsistent Location/Inconsistent Context*, (2) *Inconsistent Location/Consistent Context*, (3) *Consistent Location/Inconsistent Context*, and (4) *Consistent Location/Consistent Context*. Each binding appeared three times in each block: once as a target, and twice as a distractor. Each 36-trial block thus contained nine pseudorandomly ordered trials of each type.

We added cued-recall trials at regular intervals (i.e., every six recognition trials) within each block. In each cued-recall trial, 1 of the 36 Mandarin Chinese characters appeared in the center of the screen (see Figure 1C). Upon seeing this visual cue, participants were required to articulate the corresponding pseudoword. The target item for each of the interspersed cued-recall trials (*N* = 36) was randomly selected from one of the six preceding recognition trials. The purpose for the interspersed cued-recall trials was twofold: (1) to ensure participants were actively attempting to store the items in their memory beyond the temporal boundaries of each recognition trial, and (2) to afford participants the opportunity to practice saying the pseudowords aloud, since they would later be tested on their ability to recall the cross-modal bindings in the post-training cued-recall test. Participants were prompted to recall each binding once over the course of the task.

To familiarize the participants with the experimental procedure, training was preceded by a practice block with four recognition trials and one cued-recall trial, using additional filler stimuli. Participants were provided with feedback after each practice trial, and were given the option of repeating the practice block if needed. Participants were encouraged to take short breaks between blocks, and were instructed to resume to the same position upon their return. Re-calibration ensured that accurate eye-movements were detected following these breaks.

##### Post-training cued-recall test

As in Calabrich et al. (2021), a cued-recall test followed training. The post-training cued-recall test consisted of a single block with 36 randomly ordered trials (see Figure 1C), testing each of the previously trained bindings. A 1,000-ms fixation cross started each trial, which was then followed by a Mandarin Chinese character presented centrally for 1,000 ms in black on a white background. As in training, each character occupied 20 × 20 units of Gorilla Experiment Builder’s screen space. A 1,000-ms blank screen followed, and then a drawing of a grayscale microphone, presented in the center of the screen, indicated that the voice recording had started and a verbal response was required. Participants were instructed they would have 3 s to provide a response. However, to ensure that the onsets of participants’ responses were not inadvertently trimmed due to potential delays in the activation of the audio recording, voice recording effectively started 1,000 ms before the microphone was shown. A 250-ms blank screen then appeared, ending the trial.

##### Post-training recognition test

A single-block recognition test, comprising the same visual-auditory stimuli from the previous tasks, was administered immediately after the post-training cued-recall test. It consisted of 36 randomly ordered three-alternative forced-choice trials. These were similar to the recognition trials in the training task but lacked the encoding phase. Each trial began with a black dot presented on a white background in the center of the screen (see Figure 1D). Upon clicking on the dot, participants would hear one of the 36 target pseudowords. A 1,000-ms blank screen would follow, and three equidistant Mandarin Chinese characters would be presented in the same triangle formation as training. Participants were instructed to select the character which corresponded to the auditory cue they had just heard. A 250-ms blank screen was presented, at which point the trial ended.

### Data Analysis

To enable comparisons of eye movements across different screen sizes, we used normalized coordinates in our eye-tracking analyses wherein −0.5 and 0.5 always refer to the center of the screen regardless of their size (Gorilla Experiment Builder; Anwyl-Irvine et al., 2020). We performed fixation detection on the normalized data for each individual participant *via* the “detect.fixations” function in the “saccades” v0.2.1 library (von der Malsburg, 2019) in R v4.0.0 (R Core Team, 2020). Due to the noisier and low-frequency nature of webcam-based eye-tracking data, we set the “smooth.coordinates” parameter to “TRUE” to suppress noise, and set the “smooth.saccades” to “FALSE” to detect short saccades more reliably (von der Malsburg, 2019).

We used confirmatory logistic mixed effects regression, *via* the glmer:binomial function in the lme4 v1.1-23 library (Bates et al., 2015) in all analyses. All models included maximal random effects structures (Barr et al., 2013) reverting to a “parsimonious” approach in the case of convergence errors (Bates et al., 2015). In all models, *subject* and *item* were included as random effects. For the recognition trials from the training task—our richest source of data—we modeled error rate as a function of six fixed effects and their interactions: (1) Group membership (*Group*, i.e., typical reader = −0.5, individual with dyslexia = 0.5); (2) Context consistency (*Context*, i.e., whether a target consistently co-occurred with the same distractors over the course of the task; consistent = −0.5, inconsistent = 0.5); (3) Location consistency (*Location*, i.e., whether a target consistently appeared in the same screen location over the course of the task; consistent = −0.5, inconsistent = 0.5); (4) Repetition effects [*log(Block)*, i.e., Blocks 1–6; log-transformed]; (5) The presence of looking-at-nothing behavior (*FixatedAnyROI*, i.e., whether participants re-fixated any of the regions of interest (ROI) upon hearing the auditory cue; no = −0.5, yes = 0.5); and (6) Primary fixation (*PrimaryFixation*, i.e., the dominant region of interest fixated upon hearing the auditory cue; target = −0.5, distractor = 0.5, none = 0.0), conceptually nested within *FixatedAnyROI*. All predictors were contrast-coded and centered. In our pre-registration of this study, we conducted a power analysis using the simR library (Green and Macleod, 2016) to estimate a sample size with sufficient power for the interaction of primary theoretical interest (Group × Context × Location). Thus, when reporting the findings below, we signpost significant higher order interactions that should be interpreted with caution.

In the cued-recall trials embedded in the training task, and in the subsequent post-training tests of cued-recall and recognition, we modeled error rate as a function of the following three factors and their interactions, as described above: (1) Group membership, (2) Context consistency, and (3) Location consistency. Cued-recall errors were defined as any mis-articulations that deviated from the correct pseudoword in at least one phoneme. Recognition errors were defined as any trial in which a participant clicked on a non-target character.

## Results

### Literacy and General Cognitive Ability Measures

Background measures for both groups are summarized in Table 1. Participants with self-reported dyslexia diagnoses scored significantly higher on the ARQ (Snowling et al., 2012) than those without such diagnoses. As a group, readers with dyslexia correctly read significantly fewer words and pseudowords than did the typical readers. Similarly, typical readers were significantly faster at naming digits and letters than readers with dyslexia. There were no significant group differences on verbal and non-verbal IQ measures, nor on forward and backward digit span measures.

**TABLE 1:** Group scores on literacy and general cognitive ability measures.

Group performance								
Test	Measure	Dyslexic *N* = 35		Typical *N* = 35		*t*	*p*	Cohen’s *d*
		*M*	*SD*	*M*	*SD*			
TOWRE	Word reading rate^*a*^	74.60	19.26	90.63	9.25	4.42	<0.001	–1.05
	Pseudoword reading rate^*a*^	41.11	11.24	53.97	7.27	5.68	<0.001	–1.35
CTOPP	RAN digits^*b*^	16.46	4.1	13.31	2.61	3.82	<0.001	0.91
	RAN letters^*b*^	17.23	4.09	13.51	2.34	34.11	<0.001	1.11
WAIS	Verbal IQ^*c*^	22.66	4.14	23.31	3.74	0.69	0.488	–0.16
WASI	Non-verbal IQ^*c*^	18.50	6.7	20.69	3.92	1.64	0.105	–0.39
ARQ	Risk of reading impairment^*d*^	23.09	5.17	13.30	5.57	7.57	<0.001	–1.82
	Forward digit span^*e*^	5.27	1.7	6.03	1.76	1.80	0.076	–0.43
	Backward digit span^*e*^	4.26	1.7	5.06	1.76	1.92	0.059	–0.46

*^*a*^Number of correctly read items within 45 s.*

*^*b*^Raw scores in seconds.*

*^*c*^Raw scores.*

*^*d*^Higher scores represent greater likelihood of reading disability.*

*^*e*^Discontinue rule: two incorrectly typed responses in a row.*

### Training

#### Recognition Task

A total of 491 (3.24%) recognition trials timed out (i.e., no mouse click was detected) and were thus excluded, leaving the 14,629 trials for the behavioral analyses summarized in Table 2. Distributed across these behaviorally valid trials, the webcam-based eye tracking technique provided a total of 900,837 eye-tracking estimates in our screen of interest. We excluded approximately 3% of these estimates (*N* = 28,080) due to suboptimal face detection values (i.e., face_conf < 0.5). The noise suppression and short saccade detection filtering excluded about 16% of the data, leaving a total of 12,145 trials (6,130 dyslexic; 6,015 typical) containing both the behavioral and eye tracking measures required for our planned analyses. In these trials, readers with and without dyslexia fixated ROIs for targets and distractors in similar proportions [χ^2^(1) = 0.02, *p* = 0.88].

**TABLE 2:** Summary of subject-weighted mean error proportions in the training recognition task and interspersed cued-recall trials, post-training recognition and cued-recall tests.

Context																	
		Consistent								Inconsistent							
		*M*				*SD*				*M*				*SD*			
		TR^*a*^	TCR^*b*^	PTR^*c*^	PTCR^*d*^	TR	TCR	PTR	PTCR	TR	TCR	PTR	PTCR	TR	TCR	PTR	PTCR
	Consistent (dyslexic)	0.173	0.567	0.225	0.679	0.132	0.209	0.213	0.203	0.227	0.744	0.241	0.753	0.112	0.226	0.173	0.232
	Consistent (typical)	0.091	0.435	0.082	0.489	0.072	0.229	0.136	0.294	0.110	0.542	0.140	0.493	0.089	0.222	0.167	0.294
	**Location**																
	Inconsistent (dyslexic)	0.191	0.676	0.171	0.673	0.128	0.196	0.200	0.222	0.244	0.621	0.216	0.716	0.138	0.208	0.190	0.245
	Inconsistent (typical)	0.107	0.520	0.104	0.466	0.085	0.212	0.155	0.223	0.123	0.430	0.098	0.428	0.098	0.230	0.126	0.273

*^*a*^Training recognition.*

*^*b*^Training cued-recall.*

*^*c*^Post-training recognition.*

*^*d*^Post-training cued-recall.*

##### Error patterns common to both groups

**FIGURE 2:**
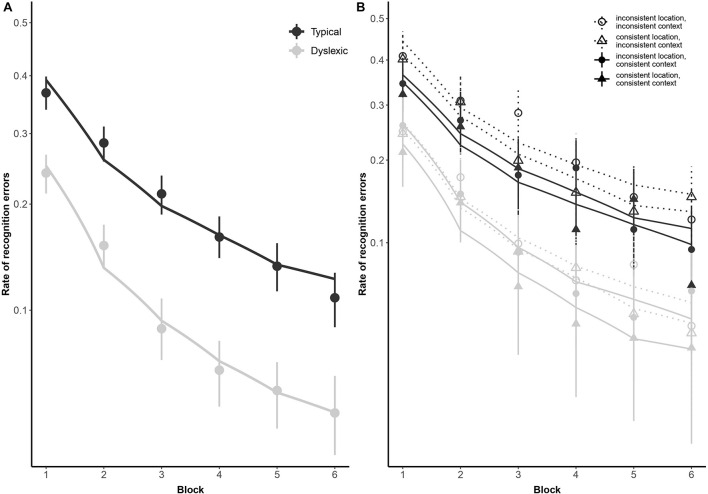
Subject-weighted mean recognition error rates as a function of reading ability and repetition in the training and recognition task. Panel **(A)** depicts overall recognition error rates for readers with dyslexia and typical readers, whereas panel **(B)** outlines the same data broken down by trial type (i.e., whether context and/or location was kept consistent during encoding). The *y*-axis is logit-scaled in both plots to match logistic regression error analyses. Point ranges represent bootstrapped confidence intervals, and lines represent logistic regression model fits.

**FIGURE 3:**
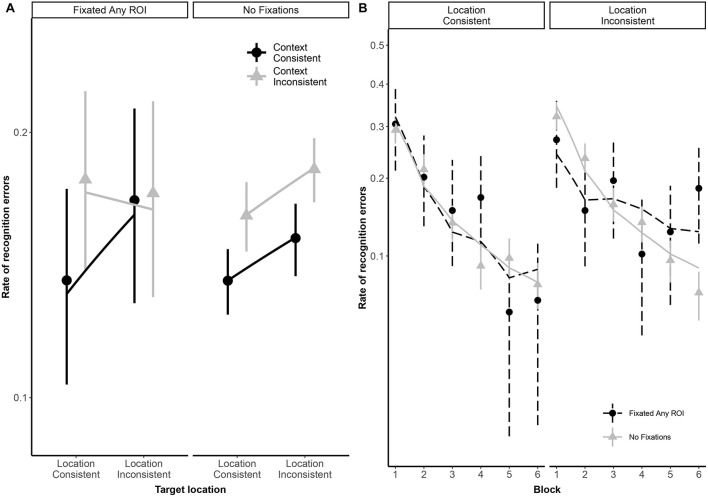
Panel **(A)** shows subject-weighted mean recognition error rate as a function of context and location consistency in trials where participants looked at any of the three regions of interest (ROI), depicted by the “FixatedAnyROI” facet, compared to trials in which looking-at-nothing behavior did not emerge. Panel **(B)** shows subject-weighted mean recognition error rate as a function of repetition (i.e., blocks) and location consistency. In both panels, the *y*-axis is logit-scaled to match logistic regression error analyses. Point ranges represent bootstrapped confidence intervals, and lines represent logistic regression model fits.

As illustrated in Figure 2, both typical readers and readers with dyslexia benefited from stimulus repetition, making fewer errors in each successive block [odds ratio: 0.32:1, β*_*log(Block)*_* = −1.13, *SE* = 0.08, *p* < 0.001]. Participants made fewer recognition errors in context-consistent conditions, when a target consistently appeared with the same distractors (odds ratio: 1.35:1, β*_*Context*_* = 0.30, *SE* = 0.13, *p* = 0.018). As illustrated in Figure 3A, participants also showed some tendency to make fewer errors in location-consistent conditions, when a target consistently appeared in the same screen location (odds ratio: 1.20:1, β*_*L*__*o*__*cation*_* = 0.19, *SE* = 0.13, *p* = 0.153), but this effect was diminished for trials in which they fixated the former location of either a target or distractor (odds ratio: 0.39:1, β_*Location* × *Context* × *FixatedAnyROI*_ = −0.94, *SE* = 0.48, *p* = 0.049). Repetition also interacted with location consistency to modulate the general looking-at-nothing effect, as illustrated in Figure 3B: when a target appeared in varied screen positions, looking at any of the three blank ROI was associated with lower recognition error rates in the early blocks, but this pattern reversed in later blocks [odds ratio: 2.33:1, β_*log(Block)* × *Location* × *FixatedAnyROI*_ = 0.85, *SE* = 0.36, *p* = 0.018].

##### Group effects

As illustrated in Figure 2, typical readers made significantly fewer errors than readers with dyslexia (odds ratio: 2.72:1, β*_*Group*_* = 1.00, *SE* = 0.22, *p* < 0.001), but there was no significant difference in how the two groups performed as a function of repetition [odds ratio: 1.30:1, β_*log(Block)* × *Group*_ = 0.26, *SE* = 0.15, *p* = 0.069]. We predicted a stronger tendency for readers with dyslexia to err more when fixating screen locations previously occupied by distractors, as previously observed by Jones et al. (2018). However, this interaction did not come out significant in our study (odds ratio: 1.08, β_*Group* × *PrimaryFixation*_ = 0.08, *SE* = 0.47, *p* = 0.864). Similarly, contrary to our prediction that spatial *and* contextual consistency would jointly decrease recognition error rates in general, albeit with a disproportionately stronger effect for readers with dyslexia, these two-way and three-way interactions also did not reach significance in the present study (odds ratio = 0.89:1, β_*Context*_ × *Location* = −0.12, *SE* = 0.26, *p* = 0.650; odds ratio = 1.18:1, β_*Group*_ × *Context* × *Location* = 0.17, *SE* = 0.29, *p* = 0.563).

**FIGURE 4:**
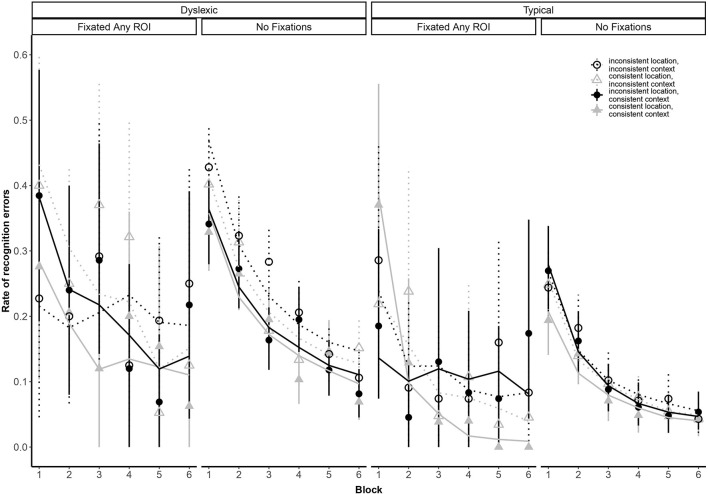
Subject-weighted mean recognition error rate as a function of repetition (i.e., blocks), group membership, context and location consistency, and whether participants looked at one of the three regions of interest (ROI). Point ranges represent bootstrapped confidence intervals, and lines represent logistic regression model fits.

Our analysis yielded a higher-order interaction involving reading ability and eye movements. Specifically, a five-way interaction between block, group, context consistency, location consistency, and ROI fixation [odds ratio: 44.78:1, β_*log(Block)* × *Group* × *Location* × *Context x FixatedAnyROI*_ = 3.80, *SE* = 1.38, *p* = 0.006; see Figure 4]. This interaction suggests differential sensitivity to presentation details, but we report it with caution because we did not anticipate the precise form of this interaction and, as noted earlier, the analysis lacks the necessary power to properly assess it (Button et al., 2013).

#### Cued-Recall Trials

**FIGURE 5:**
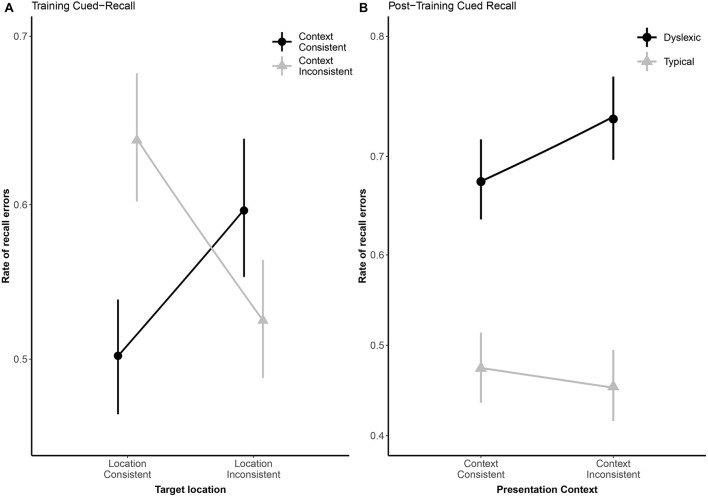
Panel **(A)** shows subject-weighted mean recall error rate as a function of context and location consistency in the cued-recall trials interspersed in training. Panel **(B)** shows subject-weighted mean recall error rate as a function of reading ability and context consistency in the post-training cued-recall test. In both panels, the *y*-axis is logit-scaled to match logistic regression error analyses. Point ranges represent bootstrapped confidence intervals, and lines represent logistic regression model fits.

Due to a playback error which rendered some of the audio files unintelligible, we excluded 82 (3.25%) of the cued-recall trials that were interspersed in the training task, leaving the 2,438 analyzable trials (1,210 dyslexic; 1,288 typical) summarized in Table 2. Overall, readers with dyslexia incorrectly recalled bindings more frequently than typical readers (odds ratio: 2.28:1, β*_*Group*_* = 0.82, *SE* = 0.19, *p* < 0.001). As illustrated in Figure 5A, location-consistency and context-consistency significantly interacted (odds ratio = 0.35:1, β_*Context* × *Location*_ = −1.04, *SE* = 0.39, *p* = 0.007), such that location-consistency only benefited recall during training when context was also consistent, but the strength of this interaction did not significantly differ between groups (odds ratio = 0.80:1, β_*Group* × *Context* × *Location*_ = −0.22, *SE* = 0.37, *p* = 0.549).

### Post-training Cued-Recall Test

We excluded 224 (8.88%) trials from the post-training cued-recall test, due to the playback error noted above, leaving the 2,296 trials (1,113 dyslexic; 1,183 typical) summarized in Table 2. Overall, readers with dyslexia incorrectly recalled bindings more frequently than typical readers (odds ratio: 3.50:1, β*_*Group*_* = 1.25, *SE* = 0.28, *p* < 0.001), but as illustrated in Figure 5B they benefited more from having consistently appeared with the same distractors during the training phase (odds ratio = 1.48:1, β_*Group* × *Context*_ = 0.39, *SE* = 0.2, *p* = 0.047).

### Post-training Recognition Test

Accuracy in the post-training recognition test is summarized in Table 2. Readers with dyslexia incorrectly recognized bindings more frequently than typical readers (odds ratio: 2.71:1, β*_*Group*_* = 0.99, *SE* = 0.33, *p* = 0.003). No other effects or interactions approached significance.

A summary with the significant effects and interactions observed in all models can be found in Table 3. A complete list with all the effects and interactions can be found in the Supplementary Material.

**TABLE 3:** Summaries of logistic mixed effects regression analyses of error frequency.

	Coef. (β)	SE (β)	*p*	OR [exp (β)]
**Recognition error frequency (training)**				
(Intercept)	−2.18	0.12	<0.001	0.11
log (Block)	−1.13	0.08	<0.001	0.32
Group (typical, dyslexic)	1.00	0.22	<0.001	2.72
Context (consistent, inconsistent)	0.30	0.13	0.018	1.35
log (Block) × Location × FixatedAnyROI	0.85	0.36	0.018	2.33
Location × Context × FixatedAnyROI	−0.94	0.48	0.049	0.39
log (Block) × Group × Location × Context × FixatedAnyROI	3.80	1.38	0.006	44.78
**Cued-recall error frequency (training)**				
(Intercept)	0.33	0.13	0.011	1.39
Group (typical, dyslexic)	0.82	0.19	<0.001	2.28
Location × Context	−1.04	0.39	0.007	0.35
**Cued-recall error frequency (post-training)**				
(Intercept)	0.44	0.16	0.007	1.56
Group (typical, dyslexic)	1.25	0.28	<0.001	3.50
Group × Context	0.39	0.19	0.047	1.48
**Recognition error frequency (post-training)**				
(Intercept)	−2.23	0.18	<0.001	0.11
Group (typical, dyslexic)	0.99	0.33	0.003	2.71

### Response Times

Although our predictions and power analyses concerned only accuracy data, for completeness, we also ran an analogous analysis of the response time data, reported in the Supplementary Material. In sum, although readers with dyslexia were generally slower at recognizing the bindings during training, response times for the accurate responses did not significantly differ between the two groups. In the post-training recognition test, however, typical readers accurately recognized the bindings significantly faster than readers with dyslexia.

## Discussion

Efficient cross-modal binding (e.g., mapping letters to letter sounds) is fundamental in the initial stages of literacy acquisition (Seidenberg and McClelland, 1989; Harm and Seidenberg, 1999), and this skill appears to be impaired in children and adults with developmental dyslexia (Blau et al., 2009; Jones et al., 2013b, 2018). Here, we examined whether dyslexic readers’ ability to track stimulus consistencies across multiple exposures might contribute to their impaired audiovisual learning (relative to typical readers), more generally considering the contributions of statistical learning and associated episodic memory processes to the acquisition of novel cross-modal bindings. Our experiment simulated the incremental process of letter-sound acquisition by repeatedly presenting participants with arbitrary visual-phonological associations. We were primarily motivated by (1) the specific question of how episodic memory cues, such as consistent spatial and contextual properties, might modulate readers’ acquisition of these novel bindings, and (2) more generally identifying differences in the learning characteristics of typical and dyslexic readers. This section is structured according to these objectives. To briefly summarize our main findings, we show that whilst all participants used stimulus consistencies in order to improve learning, readers with dyslexia may show a particular reliance on stimulus co-occurrence.

### How Statistical Consistencies Impact Cross-Modal Binding for All Participants

We examined the extent to which reliance on the consistency (or inconsistency) of spatial and contextual stimulus properties—presented across multiple exposures and trials—modulated binding performance. These effects were examined in the context of the main training task, but also in the recognition and recall post-tests. We also examined the extent to which participants would execute looks toward relevant blank screen locations previously occupied by targets, and their effect, if any, on recognition accuracy during the training task.

During training, all participants benefited from a target’s repeated presentation as part of the same three-stimulus set (i.e., context consistency; see El-Kalliny et al., 2019). Moreover, context interacted with location and screen fixations to modulate error rates: whilst *inconsistent* contexts were overall detrimental to recognition (see above), recognition accuracy in these trials nevertheless improved in location-consistent trials, in which items were consistently presented in the same screen location. However, this pattern was predominantly observed in trials where participants *did not* fixate any of the relevant ROI. We suggest that since relevant spatial information had presumably already been encoded along with the bindings, re-fixating the empty screen locations in search of spatial retrieval cues may have been redundant, or even deleterious to performance.^2^ This relationship is further modulated by stimulus repetition: recognition for stimuli presented in *inconsistent* screen locations was found to be more accurate when participants did fixate relevant screen locations, but only during the initial exposures to these stimuli (reflected in performance on the earlier blocks). However, this pattern reversed as a function of block: participants eventually became less accurate following a fixation to a relevant screen location, following multiple exposures to the stimuli. For stimuli with inconsistent locations, therefore, attempts to use spatial location as a retrieval cue became increasingly—and perhaps unsurprisingly—error prone.

In the cued-recall trials interspersed in the training task, participants from both groups also exhibited lower error rates for items consistently encoded in fixed locations *and* with fixed contexts. We speculate here that, while participants were still being trained on the novel bindings, availability of *multiple* episodic memory cues supported recall. In the absence of cues, however, or when only one consistent cue was present, recall became more effortful, and thus less accurate.

Taken together, these findings show that *all participants*, *both typical and impaired*, readily leveraged temporal and spatial consistencies to bootstrap audiovisual learning over multiple exposures. Our findings are in line with the regularity principle of statistical learning (Perry et al., 2010; Vlach and Sandhofer, 2011; Twomey et al., 2014), in which the cognitive system structures inherent environmental variability by integrating frequently occurring items by their co-occurrence, or consistency. This enables us to build supraordinate categories for words, and parts of words in the lexicon, and associated semantic webs. In real-world learning contexts, both spatial location and context would presumably be considerably more varied (though perhaps context less so), so the regularity principle would lead beginning readers to average them out as noise. When we increased the consistency of these features, however, readers appear to have incorporated these co-occurrences into their proto-orthographic representations, thus reinforcing our previous claim that even experienced readers track such information as potentially meaningful (Jones et al., 2018).

### Differential Stimulus Consistency Effects on Typical and Dyslexic Readers

Typical readers were more accurate than readers with dyslexia in all tasks, as in Jones et al. (2018) cued-recall study. The main recognition task also suggested differences in the effect of stimulus consistencies on typical and dyslexic readers’ performance, in the form of a significant 5-way interaction. Such high-order interaction is challenging to interpret, and based on pre-experiment simulations, we did not expect to have power to accurately assess them. As others have noted (e.g., Button et al., 2013), low power increases the likelihood of false positives as well as false negatives in null hypothesis statistical testing. At present, we tentatively suggest this interaction may be understood as suggesting global differences emerging for errors that implicate re-fixations vs. errors that proceed *via* direct access.

In the post-training recognition and cued-recall tests—the two tasks we administered to examine longer-term retention of the bindings—participants from the two reading groups recognized more bindings than they recalled, consistent with the general trend whereby recognition of previously studied items is often successful even when the items cannot be accurately recalled (Tulving, 1982). Overall, typical readers recognized and recalled twice as many bindings as did readers with dyslexia. We suggest that, given dyslexic readers’ propensity to benefit less from multiple exposures during training (Ahissar, 2007), there are knock-on effects for later retrieval. Their comparatively worse performance in the two post-training tests is consistent with previous studies showing reduced long-term memory capacity in readers with dyslexia (Menghini et al., 2010; Huestegge et al., 2014).

In the post-training tests, one might reasonably predict that if repeated exposure to bindings is sufficient for participants to build strong representations to support recognition and recall, they may no longer rely on episodic cues to aid memory retrieval. Behavioral data showed that whilst this was indeed the case for the typical reader group, it was not the case for readers with dyslexia: compared to typical readers, they more frequently correctly recalled bindings which had *consistently* been trained with the same distractors. We suggest that dyslexic readers’ reliance on episodic cues may be indicative of a more fragile memory representation: bindings that are robustly represented in memory are accessed and retrieved *via* a direct visual-to-auditory route rather than *via* an indirect route that is dependent on seemingly irrelevant episodic cues (Jones et al., 2018). Our findings suggest that readers with dyslexia use context in order to support retrieval, consistent with previous findings, in which dyslexic readers benefited from item presentation in a fixed temporal order (Saffran et al., 1996; Toffalini et al., 2018).

Taken together, our findings with respect to group differences show a deficit for readers with dyslexia in both recognizing and recalling audiovisual bindings of novel items, in all tasks. This finding is in line with previous PAL studies (Messbauer and de Jong, 2003; Warmington and Hulme, 2012; Jones et al., 2013b, 2018; Litt and Nation, 2014; Wang et al., 2017; Toffalini et al., 2018, 2019). Even at the behavioral level, then, adult readers with dyslexia required substantially more repetition in order to achieve accuracy comparable to typical readers (see Figure 2), a pattern that is remarkably consistent with Saffran et al. (1996) predictions that word learning in individuals with language disorders requires at least twice the exposure. Even these highly compensated adults with dyslexia were therefore relatively impervious to the effects of frequency on learning. Did this mean that they were insensitive to stimulus consistencies, which should, under normal circumstances, help in the statistical learning process? Our findings suggest not. Readers with dyslexia seemed perfectly able to use consistency in spatial location information to improve recall, which was on a par with the effect of location-consistency on their typically reading peers. This finding is at odds with the hypothesis that readers with dyslexia fail to use location information as a cue for cross-modal binding (cf. Jones et al., 2013b; Toffalini et al., 2018), as typical readers are shown to do (Treisman and Gelade, 1980; Treisman and Zhang, 2006). And it shows, moreover, that readers with dyslexia are in fact able to track longer-range statistical probabilities when the cues afforded across trials are highly salient and beneficial for item recognition. However, our findings showed a reader-type discrepancy in the use of context-consistency cues for item recognition: dyslexic readers’ error rates decreased disproportionately compared with typical readers’ when items were shown in a consistent context (i.e., item A appearing on each exposure with items B and C). Thus, readers with dyslexia showed an increased reliance on context consistency, suggesting that the entire episode (trial) was encoded as a whole. Previous studies have also noted a proclivity for chunking in dyslexia (Ullman and Pullman, 2015), in which memorization of whole word forms is favored over phonological decoding, leading to a disproportionate reliance on declarative memory for reading. We tentatively suggest that readers with dyslexia may use co-occurrences or consistencies to bootstrap their relative insensitivity to frequency: in a cognitive system that fails to efficiently integrate a current instance with previous exposures to that same item (Ahissar, 2007; Altmann, 2017), there may be a tendency to over-rely on episodic traces from within a single trial (as shown in the looks-at-nothing data), but also across trials (shown in an increased dependency on co-occurrences).

An important feature of this study is that testing was conducted *via* remote access to participants’ personal webcams to collect eye-tracking data. Despite the rigorous controls and procedures documented in the methods and results sections, such convenience does not come without its possible limitations and challenges. Online data collection generally raises a number of questions, such as the participant’s full capacity to understand and follow the instructions, length of task completion relative to similar in-lab studies, and the element of trust in participants’ self-reported data (such as dyslexia status, which we nevertheless mitigated to the extent that it is possible *via* objective literacy and cognitive measures). Collection of eye-tracking data *via* webcam-based eye tracking is a new and exciting method that requires highly stringent procedures in order to ensure the best possible data quality (see Bott et al., 2017; Semmelmann and Weigelt, 2018 for empirical validation of web-based eye-tracking as a suitable experimental method). Here, we took careful design considerations such as providing pictorial as well as written instructions, adding frequent attention checks to ensure participants’ computers were not left unattended mid-experiment, and enforcing an overall time limit to prevent excessively long breaks between tasks. We also employed a conservative filtering approach to exclude eye tracking estimates with low face detection values to avoid as much as possible fluctuation depending on variables such as lighting conditions and/or participants’ sitting conditions. We also calculated participants’ viewing distance, and avoided relying on fine-grained eye tracking analyses that would require sophisticated infrared technology.

## Conclusion

This study aimed to shed further light on audiovisual learning differences in typical and dyslexic readers. Our findings show that all of our participants used consistencies in the input during stimulus exposure in order to improve recognition and recall of items. However, dyslexic readers showed a persistent difficulty in integrating items in memory, and an overreliance on episodic detail in order to assist in the retrieval process. These findings may be of clinical relevance in understanding the challenges facing apparently high functioning adults. Overall, our findings provide novel evidence on dyslexic readers’ reduced ability to create abstracted representations in memory, relying instead on instance-based memory.

## Data Availability Statement

The datasets generated for this study can be found on GitHub (https://github.com/simOne3107/BindingExperimentLocationContextWebcamEyetracking).

## Ethics Statement

The studies involving human participants were reviewed and approved by the Bangor University Ethics Committee. The patients/participants provided their written informed consent to participate in this study.

## Author Contributions

SC, MJ, and GO designed the experiment, analyzed the data, and wrote the manuscript. SC programmed and conducted the experiment. All authors contributed to the article and approved the submitted version.

## Conflict of Interest

The authors declare that the research was conducted in the absence of any commercial or financial relationships that could be construed as a potential conflict of interest.

## Publisher’s Note

All claims expressed in this article are solely those of the authors and do not necessarily represent those of their affiliated organizations, or those of the publisher, the editors and the reviewers. Any product that may be evaluated in this article, or claim that may be made by its manufacturer, is not guaranteed or endorsed by the publisher.

## Footnotes

1
www.prolific.co

2 Note that characters’ position was changed between encoding and testing phases in 2/3 of all trials during training, to avoid strategic responses. Pilot data suggested that when the positions were congruous across the two phases, participants appeared to rely more on their ability to localize the sound source (i.e., top, bottom left, and bottom right), rather than on their ability to bind the sound to its corresponding visual stimulus, pushing performance to ceiling during the main task. Also note that no interactions with “location” approached significance in our response time analyses (see Supplementary Material), suggesting that participants were not particularly slower or faster as a function of location consistency.
