# Supplementary material for: Audiovisual Learning in Dyslexic and Typical Adults: Modulating Influences of Location and Context Consistency
Source: Front Psychol. 2021 Oct 28;12:754610. doi: 10.3389/fpsyg.2021.754610 (PMC8581559; doi:10.3389/fpsyg.2021.754610)
Supplement: Supplementary file 1 [file Data_Sheet_1.PDF]

Supplementary Material

1 Supplementary Data

1.1 Response time data

For completeness, we present the response time analyses which were not part of our a priori hypotheses. In a linear mixed effects regression analysis, log transformed response times were analysed as a function of group membership, repetition (i.e., Block), location consistency, context consistency, fixations to any regions of interest, and primary fixation (see summary in Supplementary Table 1) for the training and recognition task. We also ran a similar analysis on log transformed response times as a function of group membership, location consistency, and context consistency for the post-training recognition test (see summary in Supplementary Table 2). These analyses exclude incorrect responses as well as response times of less than 100ms; *p*-value estimations use the Wald approximation method.

1.1.1 Training

Overall, readers from both groups responded faster as a function of repetition (-50 ms; $\beta_{log(Block)}$ = -0.050, p <.001) in the training and recognition task. In general, while participants responded slower over time for items encoded under the *in*consistent context condition (26ms; $\beta_{log(Block)}$ = 0.026, p = .018), that effect was stronger for readers with dyslexia (52ms; $\beta_{Group\ x\ log(Block)\ x\ Context}$ = 0.052, p = .016).

Supplementary Table 1

Summary of a linear mixed effects regression analysis of log-transformed recognition response times (Training). Millisecond effect estimates are generated by back-transforming the effect at the intercept.

	Coef (β)	Lower (β)	Upper (β)	*p*	*in ms*
(Intercept)	7.470	7.433	7.507	-	1754.61
Group (typical, dyslexic)	-0.001	-0.062	0.060	.976	-1
Location (consistent, inconsistent)	0.037	-0.007	0.081	.097	37
Context(consistent, inconsistent)	0.020	-0.024	0.063	.376	20
FixatedAnyROI (no,yes)	0.005	-0.017	0.026	.673	5
PrimaryFixation (target, distractor)	0.005	-0.029	0.039	.775	5
log(Block)	-0.050	-0.076	0.024	<.001	-50
Group x Location	-0.002	-0.027	0.022	.863	-2
Group x Context	-0.017	-0.039	0.005	.121	-17
Group x FixatedAnyROI	0.023	-0.019	0.066	.279	23
Group x PrimaryFixation	0.029	-0.039	0.098	.401	29
Group x log(Block)	0.016	-0.035	0.067	.548	16
Location x Context	-0.021	-0.108	0.066	.638	-21
Location x FixatedAnyROI	-0.001	-0.035	0.034	.973	-1

Location x PrimaryFixation	0.008	-0.057	0.073	.814	8
Location x log(Block)	-0.017	-0.039	0.005	.122	-17
Context x FixatedAnyROI	0.009	-0.030	0.047	.658	9
Context x PrimaryFixation	-0.008	-0.073	0.058	.820	-8
Context x log(Block)	0.026	0.004	0.048	.018	26
FixatedAnyROI x log(Block)	-0.011	-0.055	0.032	.613	-11
PrimaryFixation x log(Block)	-0.031	-0.094	0.032	.329	-31
Group x Location x Context	0.013	-0.031	0.056	.561	13
Group x Location x FixatedAnyROI	0.065	-0.004	0.135	.063	65
Group x Location x PrimaryFixation	-0.031	-0.161	0.099	.642	-31
Group x Location x log(Block)	0.002	-0.041	0.044	.944	2
Group x Context x FixatedAnyROI	-0.057	-0.134	0.020	.148	-57
Group x Context x PrimaryFixation	0.075	-0.055	0.204	.260	75
Group x Context x log(Block)	0.052	0.010	0.094	.016	52
Group x FixatedAnyROI x log(Block)	-0.008	-0.095	0.078	.847	-8
Group x PrimaryFixation x log(Block)	-0.029	-0.159	0.101	.660	-29
Location x Context x FixatedAnyROI	0.007	-0.061	0.075	.834	7
Location x Context x PrimaryFixation	0.002	-0.127	0.131	.976	2
Location x Context x log(Block)	-0.010	-0.059	0.039	.683	-10
Location x FixatedAnyROI x log(Block)	-0.034	-0.113	0.045	.396	-34
Location x PrimaryFixation x log(Block)	-0.079	-0.200	0.043	.204	-79
Context x FixatedAnyROI x log (Block)	0.005	-0.064	0.074	.879	-5
Context x PrimaryFixation x log(Block)	-0.093	-0.209	0.024	.118	-93

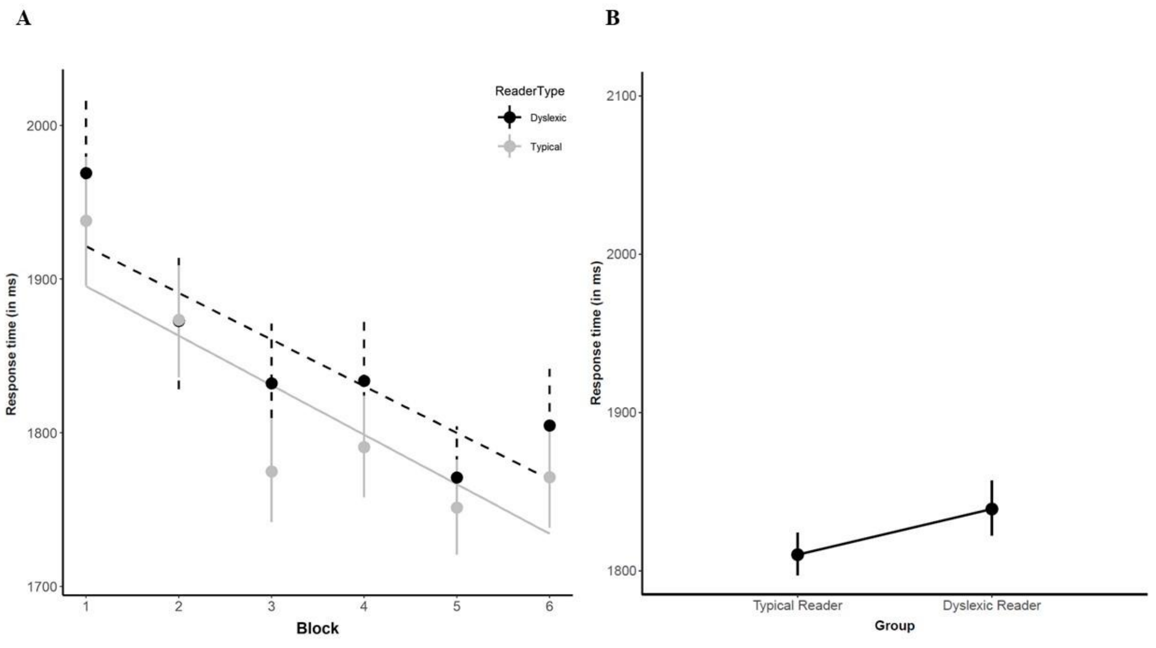

Supplementary Figure 1. Subject-weighted mean response time for accurate responses in the training and recognition task. Panel (A) shows response time data per block, while Panel (B) shows the overall response time pattern. In both plots, point ranges represent bootstrapped confidence intervals.

1.1.2 Post-training

In the post-training recognition task, readers with dyslexia responded significantly slower than typical readers (126ms; $\beta_{Group)}$ = 0.126, *p* = .015).

Supplementary Table 2

Summary of a linear mixed effects regression analysis of log-transformed recognition response times (Post-Training). Millisecond effect estimates are generated by back-transforming the effect at the intercept.

	Coef (β)	Lower (β)	Upper (β)	*p*	*in ms*
(Intercept)	7.515	7.453	7.577	<.001	1835.37
Group (typical, dyslexic)	0.126	0.024	0.227	.015	126
Location (consistent, inconsistent)	0.072	-0.005	0.149	.066	72
Context(consistent, inconsistent)	0.035	-0.041	0.111	.369	35
Group x Location	0.000	-0.058	0.058	.998	0
Group x Context	0.032	-0.023	0.086	.254	32
Location x Context	-0.133	-0.285	0.020	.088	-133
Group x Location x Context	-0.034	-0.142	0.074	.537	-34

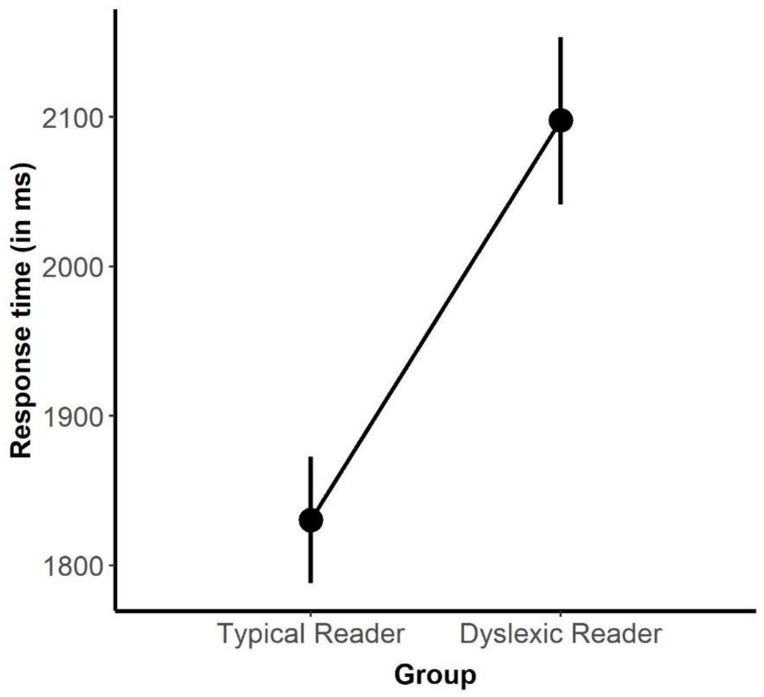

Supplementary Figure 2. Subject-weighted mean response time for accurate responses in the post-training recognition test. Point ranges represent bootstrapped confidence intervals.

1.2 Error data

Below we present the full output for each error analysis model reported in the main manuscript.

1.2.1 Training

1.2.1.1 Recognition

Supplementary Table 3

Summary of a logistic mixed effects regression analysis of recognition error frequency (Training)

	Coef(β)	SE(β)	*p*	OR (exp (β))
(Intercept)	-2.18	0.12	<.001	0.11
log(Block)	-1.13	0.08	<.001	0.32
Group (typical, dyslexic)	1.00	0.22	<.001	2.72
Location (consistent, inconsistent)	0.19	0.13	.153	1.20
Context (consistent, inconsistent)	0.30	0.13	.018	1.35
PrimaryFixation (target, distractor)	0.36	0.23	.121	1.43
FixatedAnyROI (no, yes)	-0.17	0.12	.154	0.84
log(Block) x Group	0.26	0.15	.069	1.30
log(Block) x Location	0.05	0.11	.631	1.05
Group x Location	-0.09	0.15	.525	0.91
log(Block) x Context	0.05	0.12	.706	1.05
Group x Context	0.08	0.14	.575	1.08
Location x Context	-0.12	0.26	.650	0.89
log(Block) x PrimaryFixation	0.09	0.33	.783	1.09
log(Block) x FixatedAnyROI	0.19	0.19	.314	1.21
Group x PrimaryFixation	0.08	0.47	.864	1.08
Group x FixatedAnyROI	0.02	0.26	.936	1.02
Location x PrimaryFixation	-0.13	0.46	.772	0.87
Location x FixatedAnyROI	0.24	0.25	.356	1.27
Context x PrimaryFixation	-0.04	0.46	.937	0.96
Context x FixatedAnyROI	0.23	0.24	.341	1.26
log(Block) x Group x Location	-0.07	0.19	.704	0.93
log(Block) x Group x Context	-0.12	0.21	.587	0.89
log(Block) x Location x Context	-0.01	0.22	.957	0.99
Group x Location x Context	0.17	0.29	.563	1.18
log(Block) x Group x PrimaryFixation	-1.00	0.75	.183	0.37
log(Block) x Group x FixatedAnyROI	0.29	0.36	.426	1.34
log(Block) x Location x PrimaryFixation	-0.19	0.65	.775	0.83
log(Block) x Location x FixatedAnyROI	0.85	0.36	.018	2.33
Group x Location x PrimaryFixation	-0.44	0.93	.637	0.64
Group x Location x FixatedAnyROI	-0.39	0.53	.464	0.68
log (Block) x Context x PrimaryFixation	-0.36	0.65	.576	0.69
log (Block) x Context x FixatedAnyROI	0.52	0.35	.145	1.68

Group x Context x PrimaryFixation	0.52	0.93	.575	1.68
Group x Context x FixatedAnyROI	-0.24	0.51	.643	0.79
Location x Context x PrimaryFixation	0.83	0.96	.389	2.29
Location x Context x FixatedAnyROI	-0.94	0.48	.049	0.39
log(Block) x Group x Location x Context	0.06	0.38	.882	1.06
log(Block) x Group x Location x PrimaryFixation	-1.26	1.49	.399	0.28
log(Block) x Group x Location x FixatedAnyROI	-0.82	0.70	.243	0.44
log(Block) x Group x Context x PrimaryFixation	2.60	1.49	.081	13.45
log(Block) x Group x Context x FixatedAnyROI	-0.15	0.69	.824	0.86
log(Block) x Location x Context x PrimaryFixation	-0.73	1.31	.576	0.48
log(Block) x Location x Context x FixatedAnyROI	-0.55	0.71	.438	0.58
Group x Location x Context x PrimaryFixation	-2.19	1.94	.258	0.11
Group x Location x Context x FixatedAnyROI	0.66	1.01	.517	1.93
log(Block) x Group x Location x Context x PrimaryFixation	-1.92	2.99	.520	0.15
log(Block) x Group x Location x Context x FixatedAnyROI	3.80	1.38	.006	44.78

1.2.1.2 Cued-recall

Supplementary Table 4

Summary of a logistic mixed effects regression analysis of cued-recall error frequency (Training)

	Coef (β)	SE (β)	*p*	OR (exp (β))
(Intercept)	0.33	0.13	.011	1.39
Group (typical, dyslexic)	0.82	0.19	<.001	2.28
Location (consistent, inconsistent)	-0.05	0.19	.789	0.95
Context(consistent, inconsistent)	0.19	0.19	.320	1.21
Group x Location	0.02	0.18	.898	1.02
Group x Context	0.26	0.17	.144	1.30
Location x Context	-1.04	0.39	.007	0.35
Group x Location x Context	-0.22	0.37	.549	0.80

1.3 Post-training

1.3.1.1 Cued-recall

Supplementary Table 5

Summary of a logistic mixed effects regression analysis of cued-recall error frequency (Post-training)

	Coef (β)	SE (β)	*p*	OR (exp (β))
(Intercept)	0.44	0.16	.007	1.56
Group (typical, dyslexic)	1.25	0.28	<.001	3.50
Location (consistent, inconsistent)	-0.20	0.20	.334	0.82
Context(consistent, inconsistent)	0.05	0.19	.773	1.06
Group x Location	0.08	0.19	.648	1.09
Group x Context	0.39	0.19	.047	1.48
Location x Context	-0.17	0.38	.658	0.84
Group x Location x Context	-0.03	0.41	.948	0.97

1.3.1.2 Recognition

Supplementary Table 6

Summary of a logistic mixed effects regression analysis of recognition error frequency (Post-training)

	Coef (β)	SE (β)	*p*	OR (exp (β))
(Intercept)	-2.23	0.18	<.001	0.11
Group (typical, dyslexic)	0.99	0.33	.003	2.71
Location (consistent, inconsistent)	-0.17	0.20	.379	0.84
Context(consistent, inconsistent)	0.26	0.20	.191	1.30
Group x Location	-0.22	0.27	.419	0.81
Group x Context	-0.08	0.27	.761	0.92
Location x Context	-0.28	0.40	.481	0.76
Group x Location x Context	1.01	0.54	.060	2.74
